# Healthcare staff perceptions of an electronic hand hygiene monitoring system within a large university system

**DOI:** 10.1017/ash.2024.76

**Published:** 2024-07-26

**Authors:** Rachel Elliott, Emina Fetibegovic, Julie Briggs, Jana Shaw, Paul Suits, Roger Wong, Telisa Stewart

**Affiliations:** 1 Department of Public Health and Preventive Medicine, State University of New York Upstate Medical University, Syracuse, NY, USA; 2 Department of Patient Safety and Quality, State University of New York Upstate Medical University, Syracuse, NY, USA; 3 Department of Pediatrics, Department of Public Health and Preventive Medicine, State University of New York Upstate Medical University, Syracuse, NY, USA; 4 Department of Infection Prevention, State University of New York Upstate Medical University, Syracuse, NY, USA

## Abstract

**Objective:**

The acceptability of an electronic HH monitoring system (EHHMS) was evaluated among hospital staff members.

**Design:**

An electronic HH monitoring system was implemented in June 2020 at a large, academic medical center. An interdisciplinary team developed a cross-sectional survey to gather staff perceptions of the EHHMS.

**Setting:**

The survey was conducted at a public, tertiary acute care hospital.

**Participants:**

The survey included current employees and staff. 1,273 participants responded. The mean age was 44.9 years (SD = 13.5). Most of the samples were female (71%) and non-Hispanic white (83%).

**Methods:**

A survey was conducted between June and July 2021. Responses were analyzed using Stata statistical software. Multiple logistic regression models were constructed to examine factors associated with negative perceptions of the EHHMS and its radiofrequency identification (RFID) badge. Supporting qualitative analyses were performed using Atlas.ti version 9.

**Results:**

Three-quarters (75%) of respondents reported neutral to negative perceptions of the EHHMS and its associated badge. Respondents reported limited influence on HH practices. Age, campus location, length of employment, job role, and opinion on data sharing were associated with negative perceptions of the EHHMS and RFID badge. Position in a direct patient care role was associated with negative perceptions of the RFID badge.

**Conclusions:**

Perceptions of the EHHMS aligned with previous research. Identified associations provide opportunities for targeted education, outreach, and intervention to increase acceptability and uptake. Lack of acceptance is explained by poorly perceived ease of use and usefulness, as well as challenges in implementation.

## Introduction

Hand hygiene (HH) is accepted as the primary mechanism to reduce the spread of hospital-acquired infections.^1–3^ Although the behavior of hand washing is well-supported by clinical research, compliance among healthcare workers has remained low.^4–6^ To obtain accurate measures of HH compliance, hospitals have turned to electronic HH monitoring systems (EHHMS). The acceptability of EHHMS is an ongoing topic of investigation.^7–11^

The EHHMS was implemented across the institution in 2020. The institution operates 2 hospital campuses; campus A is a large academic teaching hospital and level 1 trauma center with 438 certified beds. Hospital campus B is a community-based hospital with 314 certified beds. The EHHMS is used to monitor HH compliance and complete contact tracing. The system calculates HH compliance in rooms defined as patient space by plug-in sensors. Designated hospital staff are required to wear a personally identifiable radiofrequency identification (RFID) badge that interfaces with soap and alcohol-based hand rub dispensers to detect if HH is performed. HH is expected on entry and exit from patient spaces. The RFID badge is distinct from staff identification badges and weighs 27 g.

Infection prevention and the quality department were selected to manage the EHHMS. Devices were installed on all inpatient units, excluding psychiatric units, by the technology parent company. Devices were installed in emergency departments for contact tracing purposes only. HCW badges were distributed by employee supervisors. Only infection prevention, unit managers, and employee supervisors were granted access to the performance dashboard. The system does not provide immediate feedback on HH performance.

The purpose of the current mixed-methods study was to gauge staff acceptance of the EHHMS 1 year after implementation. We conducted an institution-wide survey to determine factors associated with negative perceptions of the EHHMS. There are few similar studies assessing staff perceptions of EHHMS at this scale, especially during the coronavirus disease 2019 (COVID-19) pandemic. The identification of personal and population characteristics associated with negative perceptions of EHHMS offers opportunities for targeted intervention to improve uptake among hospital staff.

## Methods

A voluntary survey was conducted between June 14 and July 30, 2021, to assess employee’s knowledge and attitudes surrounding the EHHMS. The survey was distributed to all staff electronically (REDCap), and paper copies were provided to employees of departments who did not access the computer network. No incentives were offered for participation.

The survey consisted of 33 questions including demographic information, multiple choice, and written response questions divided into 2 sections: (1) general HH practices and (2) use and perceptions of the EHHMS. Incomplete survey responses were excluded from the analysis.

Quantitative responses were analyzed using REDCap web features and Stata statistical software version 16 with 2-tailed tests and a 0.05 significance level. Specifically, 2 separate multiple logistic regression models were constructed to examine factors associated with negative perceptions of the EHHMS and negative perceptions of wearing the system’s RFID badge; responses were collected using a 5-point Likert scale.

Supporting qualitative analysis was performed using Atlas.ti version 9. Investigators (RE, EF, TS) independently reviewed and coded participant responses. Emerging themes were identified. One hundred percent intercoder agreement was achieved.

This project was reviewed by the Upstate Medical University Institutional Review Board (IRB). The IRB determined it did not meet the definition of human subject research under project number 1775739-1.

## Results

A total of 2,087 survey responses were received. Eight hundred fourteen incomplete responses were excluded from the analysis (n = 1,273). The survey response rate was 13.5% based on the total number of employees within the institution at the time of the survey.^12^

### Characteristics of survey respondents

The mean age of respondents was 44.9 years (SD = 13.5). Most of the sample were female (71%) and non-Hispanic white (83%). A total of 679 respondents (53%) reported they provide direct patient care. Most respondents were registered nurses (24%), ancillary support staff (23%), administration and management (14%), and scientists or physicians (10%). Additional demographic information is presented in Table 1.

**Table 1.** Sociodemographic and employment characteristics of survey respondents

Variable	Median	Mean (SD)	N (%)
***Sociodemographic***			
Age	45.0	44.9 (13.5)	
Gender			
Male			317 (71)
Female			907 (25)
Gender neutral			5 (0.4)
Other			2 (0.2)
Prefer not to disclose			42 (3.3)
Race/ethnicity			
White			1086 (83)
Black or African American			75 (6)
Asian			56 (4)
Hispanic			45 (4)
Other			40 (3)
Highest level of education completed			
Less than high school			9 (1)
High school			233 (18)
Undergraduate			617 (49)
Graduate			409 (32)
***Employment***			
Role			
Registered nurse			307 (24)
Scientist or physician			132 (10)
Administration and management			174 (14)
Ancillary services			288 (23)
Technical staff			122 (10)
Allied health professional			71 (6)
Masters-level clinician			79 (6)
Other			100 (8)
Direct patient care			
Yes			679 (53)
No			594 (47)
Primary work location			
Campus A			800 (63)
Campus B			243 (19)
Ambulatory			56 (4)
Other			164 (13)
Prefer not to disclose			10 (1)
Length of employment			
Less than 1 year			158 (12)
1–5 years			399 (31)
6–10 years			230 (18)
Greater than 10 years			486 (40)

### General HH practices

Most respondents (77%) reported performing HH at all appropriate times. Overall, staff were confident in their HH skills (98%) and felt that HH was very important (99%). The majority (86%) reported being extremely well-informed about the institution’s HH policies. Respondents were asked how HH fits into their daily work routine; over half (59%) reported HH fit extremely well.

### Electronic HH monitoring system (EHHMS)

Half of the included respondents worked in a unit or department where the electronic monitoring system was used (n = 632). The RFID badge was worn by 46% of total respondents for at least some of their working hours (n = 584). Familiarity with the system was limited (38% good; 21% moderate; 41% little). Most respondents who reported not wearing an RFID badge worked in excluded units or departments or nonhospital settings. Those working in units or departments that used the system, who elected not to wear the badge, reported that the badge was too heavy (4%), disrupted their workflow (3%), or was not a part of their daily routine (16%).

#### Staff perceptions of the system (EHHMS) and of wearing the RFID badge

Respondents who worked in a unit or department that utilized the system or wore a badge for at least some of their working hours (n = 669) expressed neutral (38%) to negative (37%) sentiments toward the system. The same was true for respondents’ sentiments toward wearing the RFID badge. Nearly half (48%) did not feel the system captured HH events accurately. Respondents were concerned about the impact of system error, user error, use of dispensers without EHHMS sensors, and additional time and effort required to ensure accurate data capture.

#### HH feedback and system impact

Most respondents who worked in a unit or department that used the system or wore a badge for at least some of their working hours reported it had little to no influence on their HH habits (54%). There was a strong desire to better understand how EHHMS data was used by the institution. Respondents reported neutral opinions about individual HH data being shared with colleagues.

Less than half of those who worked in a unit or department that used the EHHMS had received feedback on their HH performance from their manager or supervisor (43%). Supporting qualitative analysis among those who received feedback on their EHHMS HH performance indicated that the system brought HH to the forefront of thought processes and conversations, created positive and negative behavior changes, generated negative feelings, and caused concerns about data accuracy.

#### System strengths and weaknesses

Respondents who worked in a unit or department that utilized the system or who wore a badge for at least some of their working hours were asked to identify the strengths and weaknesses of the EHHMS. Four hundred nine responses were received to a question about the strengths of the EHHMS. Sixteen responses were removed because they were not codable (n = 393). Strengths identified included infectious disease control and contact tracing abilities (9.4%), as well as documentation (8.7%), accountability (7.9%), and prompts (6.1%) for HH practice. Many perceived that the system increased the mindfulness of HH (27%) (Table 2).

**Table 2.** Perceived strengths of the electronic HH monitoring system as reported by survey respondents

Theme	Description	*n* (%)
***Perceived strengths***		273 (69.5%)
Accountability	Feelings of being held accountable	31 (7.9%)
Perceptions of accuracy	System is accurate	5 (1.3%)
Perceptions of efficiency	System is efficient	9 (2.3%)
Generates positive feelings	Generates positive feelings in myself and/or other staff members	12 (3.1%)
Infectious disease control/tracing	System is helpful for infectious disease control and tracing	37 (9.4%)
Mindfulness of hand Hygiene	Increases mindfulness in myself and/or in others	106 (27.0)
Prompt for HH	Prompts myself and/or others to practice HH	24 (6.1%)
HH Feedback	Provides HH feedback	15 (3.8%)
Documentation	Documents HH	34 (8.7%)
***Generates negative feelings***	Generates negative feelings in myself and/or other staff members	10 (2.5%)
***No strengths***	System has no strengths	95 (24.2%)
***Other***	…	15 (3.8%)

Note. HH, hand hygiene.

Four hundred thirty-eight participants responded when asked about the weaknesses of the EHHMS. Twenty-six responses were removed because they were not codable (n = 412). The main system weakness identified was the perceived inaccuracy of data (37.4%). Other weaknesses included the potential to monitor staff location (12.9%) and technological problems with the RFID badge (12.4%). The presence of inactive or broken sensors was identified as a system limitation (10.4%). Some participants felt the system generated negative behaviors and feelings, and staff expressed interest in improved feedback and resource allocation decisions (Table 3).

**Table 3.** Perceived weaknesses of the electronic HH monitoring system as reported by survey respondents

Theme	Description	*n* (%)
***Perceived system weaknesses***		316 (76.7%)
Limited feedback	Limited feedback provided to me and/or other staff members	9 (2.2%)
Inactive/broken sensors	Inactive and/or broken sensors	43 (10.4%)
Perceptions of inaccuracy	System is inaccurate	154 (37.4%)
Waste of resources	System is a waste of resources	6 (1.5%)
Monitoring concerns	Monitoring concerns regarding myself and/or other staff members	53 (12.9%)
Badge concerns	RFID badge concerns	51 (12.4%)
***Perceived self and employee Weaknesses***		66 (16.0%)
Generates negative Behaviors	Generates negative behaviors in myself and/or other staff members	10 (2.4%)
Generates negative Feelings	Generates negative feelings in myself and/or other staff members	56 (13.6%)
***No weaknesses***	System has no weaknesses	16 (3.9%)
***Other***	…	14 (3.4%)

Note. HH, hand hygiene; RFID, radiofrequency identification.

#### Factors associated with negative perceptions of the EHHMS

A multiple logistic regression model was constructed to determine factors associated with negative perceptions of the EHHMS among those who worked in a unit or department that utilized the EHHMS (n = 577). Respondents of older age had more positive perceptions of the EHHMS than those who were younger (OR = .95, 95% CI .92, .97, *P* < .001). Other major demographic characteristics were not significantly associated with system perceptions (Table 4).

**Table 4.** Multiple logistic regression results for factors associated with negative perceptions of the EHHM system

Variable	OR	95% CI	*P* value
***Sociodemographic***			
Age	.95	.92, .97	<.001
Gender (reference: female)			
Male	1.13	.66, 1.95	.648
Other	5.13	.35, 75.93	.234
Race/ethnicity			
White, non-Hispanic	Reference	Reference	Reference
Black, non-Hispanic	.43	.16, 1.13	.87
Hispanic	.72	.22, 2.37	.594
Asian	.46	.16, 1.28	.135
Other	1.52	.32, 7.23	.595
Highest level of education completed			
Less than high school	Reference	Reference	Reference
High school or GED	.40	.021, 7.64	.546
Undergraduate	.29	.01, 5.90	.423
Graduate	.29	.01, 6.23	.432
***Employment***			
Role			
Registered nurse	16.80	2.02, 139.93	.009
Scientist or physician	16.17	1.69, 154.55	.016
Administration and management	Reference	Reference	Reference
Ancillary services	3.71	.41, 33.84	.246
Technical staff	10.74	.40, 291.80	.159
Allied health professional	9.13	.99, 84.33	.051
Masters-level clinician	11.40	1.27, 102.12	.030
Other	9.23	.84, 101.56	.069
Direct patient care (reference: no)	1.68	.79, 3.54	.176
Primary work location			
Campus A	Reference	Reference	Reference
Campus B	2.72	1.65, 4.47	<.001
Ambulatory	.81	.13, 7.45	.990
Length of employment			
Less than 1 year	Reference	Reference	Reference
1–5 years	3.09	1.49, 6.42	.002
6–10 years	6.99	3.09, 15.84	<.001
Greater than 10 years	6.66	2.79, 15.89	<.001
***HH***			
Policy familiarity			
Not at all	Reference	Reference	Reference
Not very	2.08	.13, 34.45	.53
Somewhat	1.27	.50, 3.19	.44
Extremely	1.93	1.21, 3.08	<.01
Perception of seeing others’ HH data			
Very negative	Reference	Reference	Reference
Negative	.51	.24, 1.09	.07
Neutral	.23	.12, .44	<.001
Positive	.07	.03, .20	<.001
Very positive	.01	.00, .12	<.001

Note. EHHM, electronic HH monitoring; OR, odds ratio; CI; confidence interval; HH, hand hygiene.

Individuals working at campus B had significantly greater odds of having a negative perception of the EHHMS than those who worked primarily at campus A (OR = 2.72, 95% CI 1.65, 4.47, *P* < .01). Job role was strongly associated, especially among individuals employed in roles outside of administration and management. Registered nurses (OR = 16.80, 95% CI 2.02, 139.93, *P* = .01), scientists and physicians (OR = 16.17, 95%CI 1.69, 154.55, *P* = .02), and masters’ level clinicians (OR = 11.40, 95% CI 1.27, 102.12, *P* = .03) had the greatest odds of expressing negative perceptions of the EHHMS. In addition, those who reported employment at the institution for greater than 1 year had much higher odds of negative perceptions than those employed for less than 1 year (Table 4).

To assess the contribution of HH knowledge and familiarity with institutional policy, these factors were included in the multiple logistic regression model. Compared with those who were not familiar with the institution’s HH policies, the odds of negative perceptions were significantly greater among those who were familiar with the policies (OR = 1.93, 95% CI 1.21, 3.08, *P* = .02). Opinion of data sharing was included as well. Those who expressed more positive opinions about individual HH data being shared with colleagues had lower odds of perceiving the system negatively than those who were strongly against the sharing of data (Table 4).

#### Factors associated with negative perceptions of wearing the EHHMS RFID badge

A second multiple logistic regression model was constructed to determine factors associated with negative perceptions of wearing the EHHMS RFID badge. This analysis included only those who reported wearing a badge for at least some of their working hours (n = 542). Self-identification of race as ‘Other’ was significantly associated with negative perceptions of wearing the badge (OR = 6.82, 95% CI 1.29, 36.04, *P* = .02). The remaining demographic results were similar to those reported above for system perceptions; older respondents remained more likely to perceive the system positively than younger respondents (OR = .97, 95% CI .95, .10, *P* = .02).

Job role and primary work location were associated with negative perceptions of wearing the RFID badge as well. Respondents working at campus B had nearly 2 times the odds of perceiving badge wearing negatively, compared with those working primarily at campus A (OR = 1.88, 95% CI 1.15, 3.05, *P* = .01). Those employed as registered nurses, scientists or physicians, and masters’ level clinicians continued to have the greatest odds of negative perceptions (Table 5). The odds of negative badge perceptions also increased with longer terms of employment; odds were greatest among those employed between 6 and 10 years (OR = 4.55, 95% CI 2.05, 10.06, *P* < .001) and those employed greater than 10 years (OR = 3.63, 95% CI 1.58, 8.35, *P* < .001). Respondents with negative perceptions of data sharing were also more likely to have negative perceptions of wearing the RFID badge, compared to those who reported neutral (OR = .32, 95% CI .16, .62, *P* = .001), positive (OR=.05, 95% CI .12, .17, *P* < .001), or very positive (OR = .02, 95% CI .00, .21, *P* = 0.001) opinions on sharing of HH compliance with colleagues.

**Table 5.** Multiple logistic regression results for factors associated with negative perceptions of wearing the EHHM RFID badge

Variable	OR	95% CI	*P* value
***Sociodemographic***			
Age	.97	.95, .99	.018
Gender (reference: female)			
Male	1.12	.64, 1.92	.696
Race/ethnicity			
White, non-Hispanic	Reference	Reference	Reference
Black, non-Hispanic	.69	.25, 1.87	.464
Hispanic	.92	.29, 2.95	.891
Asian	.69	.26, 1.84	.459
Other	6.82	1.29, 36.04	.024
Highest level of education completed			
Less than high school	Reference	Reference	Reference
High school or GED	.42	.03, 6.78	.544
Undergraduate	.36	.02, 6.12	.478
Graduate	.26	.01, 4.68	.358
***Employment***			
Role			
Registered nurse	16.26	1.70, 155.35	.015
Scientist or physician	17.63	1.57, 197.77	.020
Administration and management	Reference	Reference	Reference
Ancillary services	4.51	.14, 49.42	.217
Allied health professional	8.78	.84, 91.49	.069
Masters-level clinician	16.62	1.60, 172.59	.019
Other	5.87	.47, 73.75	.171
Direct patient care (reference: no)	2.66	1.28, 5.52	.009
Primary work location			
Campus A	Reference	Reference	Reference
Campus B	1.88	1.15, 3.05	.011
Ambulatory	1.11	.15, 8.11	.920
Other	.31	.03, 3.02	.315
Length of employment			
Less than 1 year	Reference	Reference	Reference
1–5 years	2.47	1.21, 5.02	.013
6–10 years	4.55	6.05, 10.06	.000
Greater than 10 years	3.63	1.58, 8.35	.002
***HH***			
Policy familiarity			
Not at all	Reference	Reference	Reference
Not very	.40	.00, 40.21	.695
Somewhat	1.00	.39, 2.57	.994
Extremely	1.15	.72, 1.83	.551
Perception of seeing others’ HH data			
Very negative	Reference	Reference	Reference
Negative	.53	.25, 1.15	.109
Neutral	.32	.16, .62	.001
Positive	.05	.02, .21	.000
Very positive	.02	.00, .21	.001
Badge location			
Lanyard	Reference	Reference	Reference
Breast pocket/upper chest	.76	.48, 1.21	.250
Waist	1.67	.71, 3.94	.240
Other	.75	.23, 2.41	.624

Note. EHHM, electronic HH monitoring; RFID, radiofrequency identification; OR, odds ratio; CI; confidence interval; HH, hand hygiene.

Provision of direct patient care was also included in the badge-wearing model because all currently badged employees are heavily integrated into the patient environment. Notably, the odds of a negative perception of wearing the RFID badge were nearly 3 times greater among those who reported providing direct patient care (OR = 2.67, 1.28, 5.52, *P* = .01).

#### Supporting staff who use the EHHMS

Feedback was collected at the conclusion of the survey on how the institution could better support staff whose unit or department utilizes the EHHMS. Responses focused on improving system function and accuracy, improving communication, or removing the system all together. Technological improvements, improved accuracy, open acknowledgment of system limitations, and improving access to data were discussed in open-ended responses. It was also reported that providing generalized support to staff would support the use of the EHHMS.

## Discussion

Staff perceptions of the system (EHHMS) and staff perceptions of wearing the RFID badge indicate that there is room for improvement in the acceptance of the EHHMS. The neutral and negative opinions reported echo existing literature on EHHMS.^9,10^ The concerns and weaknesses identified by hospital staff align with those previously described.^7,8,13^ Overall, lower levels of acceptance are likely a result of challenges faced while implementing the EHHMS at the height of the COVID-19 pandemic.

The institution’s intent was to implement the system quickly, with minimal disruption to staff, while providing a basic understanding of EHHMS and ensuring compliance with established HH protocols. Respondent’s limited familiarity with the system indicates that this basic understanding was not widely accomplished prior to system installation. This provided a breeding ground for the establishment of negative perceptions and behaviors. Without a thorough understanding of the EHHMS function and how data was utilized by the institution, some staff opted to forego wearing the RFID badge. Staff also adopted negative behaviors associated with the EHHMS, including the removal and relocation of sensors defining patient space. These factors reduced data capture abilities and affected system accuracy. Increased communication and improved education during the pre-implementation and implementation periods would likely have decreased the establishment and tenacity of negative perceptions among staff.^13–15^

Based on our results, we hypothesize that acceptance of EHHMS aligns with the technology acceptance model.^16^ In our sample, variables separate from the EHHMS, including demographic and employment characteristics (ie, age, job role, length of employment), influenced staff perceptions of how useful the system was. Early challenges with system management negatively influenced perceived ease of use. This bolstered initial negative attitudes toward EHHMS and further reduced system use and acceptance. This model helps to account for the variation observed in negative perception by job role, as those in leadership and management may be more likely to perceive administrative benefits of using EHHMS. Leaders and managers also have less frequent daily interactions with the EHHMS, reducing the burden of behavior change compared to staff providing direct patient care.

Further investigation is needed to determine the best methods to address negative perceptions of EHHMS; however, our analyses offer a unique identification of factors associated with EHHMS technology acceptance. Negative behaviors and concerns reported by respondents identify opportunities for targeted outreach, education, and intervention that the institution plans to operationalize. Data acquired as part of this assessment is likely useful in the development of behavioral interventions to address perceptions and use of EHHMS, as well as in the identification of high-priority groups to be addressed during future implementation efforts.

This assessment does have several limitations. First, only 46% of respondents reported actively wearing an RFID badge for at least some of their working hours. This was addressed by constructing individual multiple logistic regression models among appropriate subpopulations; however, responses from those working outside of areas with the EHHMS may have influenced levels of overall awareness and knowledge. The use of a convenience sample may also introduce self-selection and nonresponse bias. We believe this stems from several factors, including increased participation among those with particularly negative perceptions of the EHHMS and increased likelihood of participation from staff highly motivated to increase HH compliance, who are more likely to participate in a monitoring program. Generalizability of the assessment is limited secondary to the large, university setting in which data was collected as well; results may not be applicable to differing environments or staff populations. Lastly, further validation of the associations discovered with age, race, job role and location, term of employment, policy knowledge, opinion of data sharing, and provision of direct patient care is required to support the novel conclusions reached.

## Supporting information

Elliott et al. supplementary material Elliott et al. supplementary material

## Supplementary material

For supplementary material accompanying this paper visit http://doi.org/10.1017/ash.2024.76. click here to view supplementary material

## Research transparency and reproducibility

Data is not publicly available.
