# Supplementary material for: Healthcare staff perceptions of an electronic hand hygiene monitoring system within a large university system
Source: Antimicrob Steward Healthc Epidemiol. 2024 Jul 26;4(1):e103. doi: 10.1017/ash.2024.76 (PMC11736453; doi:10.1017/ash.2024.76)
Supplement: Elliott et al. supplementary material [file S2732494X24000767sup001.pdf]

Supplemental Materials

Categorization of Self-Reported Job Titles

**Job Category**	**Self-Reported Job Title**
Registered Nurses	Registered Nurse
Scientists and Physicians	Faculty: Teaching and/or Research
	Medical Student
	Physician
	Research Technician
	Resident/Fellow
Administration and Management	Administration and Management
	Educational Support
Ancillary Services	Clerical
	Clinical Support Staff
	Dietary Services
	Environmental Services
	Operating Room Technician
	Phlebotomy
	Registration
	Unit Support Technician (UST)
Technical Staff	Information Management
	Laboratory Staff

	Pharmacy
	Physical Plant
Allied Health Professionals	Imaging Staff
	Respiratory Therapist
	Therapist [OT/PT/SPT]
Masters level clinicians	Dietician
	Physician Assistant/ Nurse Practitioner
	Social Worker
Public Safety and Spiritual Care	Public Safety
	Spiritual Care
Other	Other
